# Ethnobotanical survey of plant species for herbal tea in a Yao autonomous county (Jianghua, China): results of a 2-year study of traditional medicinal markets on the Dragon Boat Festival

**DOI:** 10.1186/s13002-018-0257-0

**Published:** 2018-09-05

**Authors:** Bing Jin, Yujing Liu, Jiaxi Xie, Binsheng Luo, Chunlin Long

**Affiliations:** 1 College of Sciences, Nanjing Agricultural University, Nanjing, 210095 China; 2 Ecological Institute, Jiangsu University, Zhenjiang, 212013 China; 3 Kunming Institute of Botany, Chinese Academy of Sciences, Kunming, 650201 China; 4 College of Life and Environmental Sciences, Minzu University of China, Beijing, 100081 China

**Keywords:** Herbal tea, Ethnomedicine, Yao people, Dragon Boat Festival, Quantitative methods

## Abstract

**Background:**

Herbal tea is widely consumed in Jianghua, a Yao autonomous county in Hunan Province, China, to prevent and treat diseases. The materials in herbal tea at the traditional medicinal markets at the Dragon Boat Festival remain unknown. The aims of the paper were (1) to specifically investigate the materials of herbal tea used by Yao nationalities in Hunan Province, (2) to record the most common and the culturally important medicinal plant species in the markets, and (3) to compare the medical plant tradition both used for herbal tea between the Jianghua and Lingnan regions.

**Methods:**

During 2016–2017, 215 vendors were interviewed at traditional medicinal markets at the Dragon Boat Festival in Jianghua to record plants used for herbal tea and to document traditional knowledge of their medicinal function, habitat, and conservation status. Bunches of medicinal plants were purchased to identify the species and to prepare voucher specimens. Cognitive salience (CS) based on free-lists and use value (UV) were calculated to analyze the cultural importance of medical plants; other quantitative methods (coefficient of similarity and chi-square analysis) were applied for comparison of herbal tea tradition between the Jianghua and Lingnan regions.

**Results:**

A total of 169 species belonging to 66 families and 142 genera were recorded in herbal tea to treat health conditions in the study area. There were 30 health conditions that were recorded, with heat-clearing and detoxifying being the most common medicinal function, followed by treating rheumatism and promoting blood circulation. Of the 169 species, 97 were herbs. The whole plant was the most commonly used plant part in the preparation of herbal tea. According to the national evaluation criteria, three of these species are listed on “China’s red list” and registered as vulnerable (VU). By comparing the coefficient of similarity of herbal tea plants and the number of mentions for part(s) used in Jianghua and Lingnan, the medicinal plant tradition is different in two areas.

**Conclusions:**

Herbal tea in Jianghua reflects the cultural diversity of the Yao people and the plant diversity of the region. Future research on the safety, efficacy, and the adulterants of herbal tea are needed for sustainable utilization.

## Background

The practice of drinking herbal tea is an ancient custom for Yao people. Herbal tea is produced from water infusions of a range of plant species other than *Camellia sinensis* (L.) O. Ktze. [1]. Plant material may consist of fresh or dried parts from a single species or from multiple species. For millennia, the Yao people have been famous for being good at identifying herbs [2]. However, no documentary records have survived from when Yao medicine originated.

The Yao nationality of China is mainly distributed in Guangxi, Hunan, Guangdong, Yunnan, Guizhou, and Jiangxi Provinces. The Yao people from Hunan Province are the birthplace of the Yao nationality; Jianghua Yao Autonomous County has the largest Yao population in Hunan Province, accounting for 53% of the population there [2]. Thus, Jianghua Yao Autonomous County plays an important part in the medicine and culture of the Yao people. For historical reasons, the Yao people live long in adverse circumstances, and in the long struggle against disease, the local people had to collect herbs from surrounding mountains and valleys, and they made herbal tea to treat associated health conditions. This tradition formed different, plentiful, and special medical customs, especially herbal tea and medicated baths.

The traditional medical market is a unique custom to celebrate the Dragon Boat Festival (May 5 in the Chinese lunar calendar) by Yao, Zhuang, and Han people in Jianghua (mostly Yao people). At every Dragon Boat Festival, people collect herbs from surrounding mountains and valleys and sell them at the medical market, which is a large-scale market, with more variety and larger trades. The traditional medical market has become a unique spectacle of Yao medicinal culture customs. In addition to buying and selling various herbs, people take this opportunity to exchange their experiences in the recognition and usage of herbs. Since the Dragon Boat Festival is at the end of spring and the beginning of summer, weather conditions are volatile and moist, which probably contribute to the disease rate. During this time, many Chinese herbal medicines are in the periods of harvesting or barking, so the timing forms the unique medicinal market of Yao nationality in Jianghua.

The traditional knowledge of herbs is the result of the accumulated experience by the Yao people’s long-term struggle against disease; thus, many aspects of these treatments are probably scientific. However, like the loss of biodiversity, due to the influence of foreign culture, and not having their own written languages, with descendants inheriting their knowledge just by dictation, the traditional knowledge and culture of Yao medicine is also in danger of being lost. In fact, the vanishing of traditional knowledge has been a common phenomenon in the undeveloped country [3].

In order to protect the traditional knowledge of Yao medicine, guarantee food safety, and meet the increasingly globalized health supplement market, we started to document, explore, and research the herb materials for the preparation of herbal tea in Jianghua in 2016.

The study aims to not only document plant species used and commercialized as herbal tea in Jianghua but also make a comparison of herbal tea tradition between the Jianghua and Lingnan regions. This is the first study to document the plant species used as herbal tea in Jianghua; the medicinal plant tradition was recorded for future investigations and policy-making. As well as, if these plant materials are classified and used correctly, the opportunity to develop Yao medicine and expand the herbal tea culture will emerge.

## Methods

### Study area

**Fig. 1:**
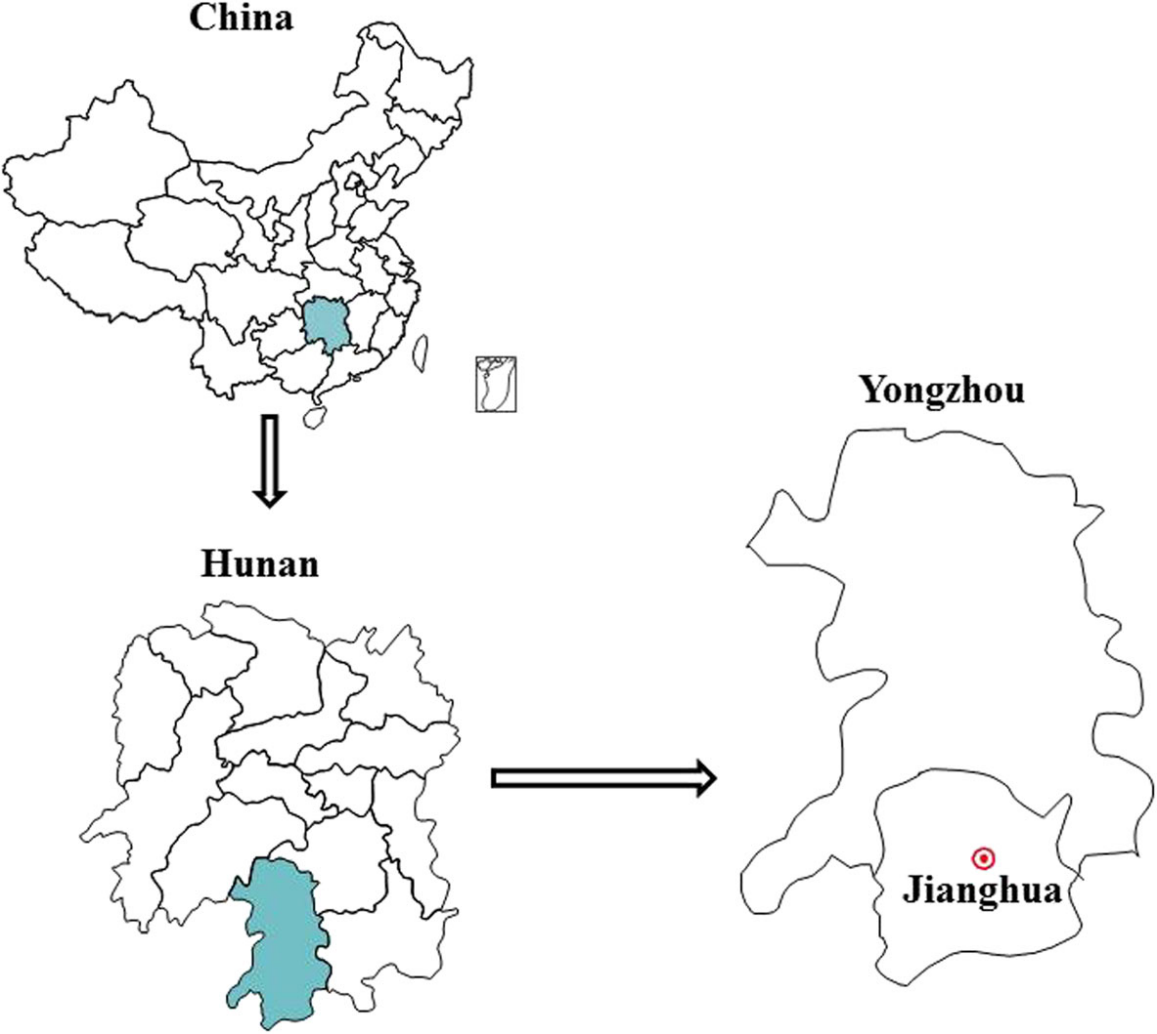
Location of the traditional medicinal market in Jianghua that was selected as a study site

The study was conducted in Jianghua, where herbal tea has a significant cultural value and it is traditionally consumed. This region is located in Yongzhou City, which borders Guangdong and Guangxi Provinces, between 110° 25′ S–112° 10′ S and 24° 38′ W–25° 15′ W (Fig. 1). It covers an area of 3248 km^2^. The total population of Jianghua was 510,000 inhabitants in 2013. It is the only Yao autonomous county in Hunan Province, with the largest population of Yao nationality in the 13 Yao autonomous counties throughout the country. This area features a subtropical monsoon climate, and the weather is relatively moderate, with an annual average temperature of 18–18.5 °C, and plenty of rainfall. It owns the biggest and most famous medicinal market in Hunan Province and the surrounding region, that is, the traditional medicinal markets at the Dragon Boat Festival.

### Traditional medicinal markets at the Dragon Boat Festival

The Dragon Boat Festival, or known as the Duanwu Festival, is a traditional Chinese cultural holiday. The festival occurs on the 5th day of May in the traditional Chinese calendar. There are three most well-known and widespread activities conducted to celebrate the Dragon Boat Festival, preparing and eating *zongzi*, drinking realgar wine, and dragon boat racing. These customs could be dated back to over 2500 years ago [4]. The Dragon Boat Festival was held at the summer solstice which is a period of high incidence of disease. Many Chinese folklorists pointed out that the Dragon Boat Festival originated from the concept of people fighting diseases and exterminating evils [5, 6]. So, during the Dragon Boat Festival, some indigenous persons, country doctors, and herbalists collect various kinds of plant and sell them to customers, retailers, or formal vendors at the traditional medicinal market.

### Ethnobotanical methods

Field surveys including informant interview, structured investigation, free-listing tasks, and voucher specimen collection were conducted during the Dragon Boat Festival in 2016 and 2017. A total of 215 vendors between 22 and 83 years of age were interviewed at the traditional medicinal markets at the Dragon Boat Festival in Jianghua, Hunan Province, to record plants used for herbal tea and to document traditional knowledge on their medicinal function, habitat, and conservation status. Of the vendors, 70% were over 50 years of age, and these vendors were almost equally male and female. The study was carried out following the International Society of Ethnobiology Code of Ethics [7], and all of the participants were informed of our intent prior to the start of the interviews. In addition, every vendor signed a benefit-sharing agreement. The majority of the vendors worked independently or in small groups, and when the vendors spoke only the Yao language, translation was required by an individual that we had hired. Vendors were asked to complete structured ethnobotanical questionnaires, which were answered willingly without payment, the questions included (1) *Which species are used for herbal tea?* (2) *Where do you gather this plant?* (3) *What plant parts can be used for herbal tea?* (4) *What is the function of this plant in herbal tea?* and (5) *What plants do Yao people here use for herbal tea?* Bunches of medicinal plants were purchased to identify the species and to prepare the voucher specimens followed by the *Flora of China* (http://frps.eflora.cn/) and the collections in PE (the Herbarium, Institute of Botany, Chinese Academy of Sciences), and KUN (the Herbarium, Kunming Institute of Botany, Chinese Academy of Sciences). We then deposited them in the Ethnobotanical Lab, Minzu University of China. Photographs were taken to record all of the plant species.

The conservation status of each plant was revised by the Information System of Chinese Rare and Endangered Plants (http://rep.iplant.cn/protlist/7) (Table 1).

**Table 1:** Medicinal plants used for herbal tea in the traditional medicinal market of Jianghua County on Dragon Boat Festival

Family name	Scientific name	Chinese name	Yao name	Medicinal use	Part(s) used	Habitat	Originality	Conservation status	Use value	Frequency	Voucher number
Acanthaceae	*Andrographis paniculata* (Burm. f.) Nees	穿心莲	Chuan fin lian	Heat-clearing and detoxifying, eliminating inflammation	Whole plant	Herb	Chinese PharmacopeiaI (2015) P268	NE	1.10	16	JH-141
Aceraceae	*Acer mono* Maxim.	色木槭	Se diang qie	Rheumatism	Stem, leaf	Tree	–	NE	0.87	12	JH-021
Acoraceae	*Acorus tatarinowii* Schott	石菖蒲	Lao bie chang pu	Cold	Whole plant	Herb	Chinese PharmacopeiaI (2015) P91	NE	0.87	65	JH-202
Adoxaceae	*Viburnum odoratissimum* Ker-Gawl.	珊瑚树	Shan hu dang	Rheumatism	Whole plant	Shrub	–	NE	0.85	39	JH-035
Amaranthaceae	*Achyranthes aspera* L.	土牛膝	Tu ong che bo	Heat-clearing and detoxifying, rheumatism, nourishing, relieve pain	Whole plant	Herb	–	NE	1.51	45	JH-267
Amaranthaceae	*Achyranthes bidentata* Blume	牛膝	Ong che bo	Nourishing	Root	Herb	Chinese PharmacopeiaI (2015) p72	NE	0.85	56	JH-050
Angiopteridaceae	*Angiopteris fokiensis* Hieron.	福建观音座莲	Fu jian guan yin zuo lian	Heat-clearing and detoxifying, promote blood circulation, relieve pain	Rhizome	Fern	–	NE	1.25	18	JH-222
Annonaceae	*Fissistigma polyanthum* (Hook. f. et Thoms.) Merr.	黑风藤	Ji jia mei	Rheumatism	Whole plant	Shrub	Chinese PharmacopeiaI (1977) p593	NE	0.84	40	JH-298
Apocynaceae	*Cynanchum paniculatum* (Bunge) Kitagawa	徐长卿	Xu chang qing	Heat-clearing and detoxifying, eliminating inflammation, relieve cough	Whole plant	Herb	Chinese PharmacopeiaI (2015) p285	NE	1.25	64	JH-278
Apocynaceae	*Dischidia australis* Tsiang et P. T. Li	尖叶眼树莲	Lai nong mu jin diang lian	Eliminating inflammation, rheumatism	Whole plant	Vine	–	NE	1.09	33	JH-127
Apocynaceae	*Marsdenia sinensis* Hemsl.	牛奶菜	Ong you lai	Rheumatism, promote blood circulation, heatstroke	Stem	Vine	–	NE	1.09	35	JH-151
Apocynaceae	*Trachelospermum jasminoides* (Lindl.) Lem.	络石	Luo lao	Rheumatism	Whole plant	Vine	Chinese PharmacopeiaI (2015) p269	NE	0.84	30	JH-045
Aquifoliaceae	*Ilex chinensis* Sims	冬青	Dong men	Promote blood circulation	Bark, seed	Tree	Chinese PharmacopeiaI (1977) p107	NE	0.84	41	JH-182
Araceae	*Pothos chinensis* (Raf.) Merr.	石柑子	Lao bie gan zei	Rheumatism	Whole plant	Vine	–	NE	0.84	33	JH-185
Araceae	*Typhonium flagelliforme* (Lodd.) Blume	鞭檐犁头尖	Bian yan li tou jian	Heat-clearing and detoxifying, relieve cough	Root	Herb	–	NE	1.09	16	JH-017
Araliaceae	*Acanthopanax evodiaefolius* Franch.	吴茱萸五加	Wu zhu yu heng jia	Rheumatism	Rhizome	Shrub	–	NE	0.84	39	JH-102
Araliaceae	*Heteropanax fragrans* (Roxb.) Seem.	幌伞枫	Huang fan jia	Rheumatism	Bark, pith	Tree	–	NE	0.83	36	JH-220
Araliaceae	*Panax japonicus* (T. Nees) C. A. Mey.	竹节参	Lao a shen	Nourishing, eliminating phlegm, stop bleeding, relieve pain	Rhizome	Herb	Japanese Pharmacopoeia 17	NE	1.48	15	JH-244
Araliaceae	*Schefflera octophylla* (Linn.) Frodin	鹅掌柴	E zhuan zhan	Heat-clearing and detoxifying, rheumatism, relaxing tendons, and activating collaterals	Leaf, bark	Shrub	–	NE	1.47	40	JH-081
Aristolochiaceae	*Asarum sagittarioides* C. F. Liang	山慈菇	Geng ci jiu	Rheumatism, relieve pain	Whole plant	Herb	Chinese PharmacopeiaI (2015) p32	NE	1.09	23	JH-277
Basellaceae	*Basella alba* L.	落葵	Luo kui	Heat-clearing and detoxifying	Leaf, whole plant	Herb	–	NE	1.08	37	JH-119
Berberidaceae	*Dysosma versipellis* (Hance) M. Cheng ex Ying	八角莲	Ba guo lian	Heat-clearing and detoxifying, promote blood circulation	Rhizome	Herb	–	VU	1.08	12	JH-235
Berberidaceae	*Mahonia fortunei* (Lindl.) Fedde	十大功劳	Jie da gong luo	Heat-clearing and detoxifying	Root, stem	Shrub	–	NE	0.83	60	JH-241
Caesalpiniaceae	*Bauhinia championii* (Benth.) Benth.	龙须藤	Long xu mei	Rheumatism, relaxing tendons, and activating collaterals, relieve pain	Stem	Vine	–	NE	1.45	31	JH-285
Cannabinaceae	*Humulus scandens* (Lour.) Merr.	葎草	Lv mi	Heat-clearing and detoxifying, induce diuresis	Whole plant	Herb	–	NE	1.08	17	JH-226
Caprifoliaceae	*Lonicera confusa* (Sweet) DC.	华南忍冬	Hua nan yin dong	Heat-clearing and detoxifying	Flower, stem, leaf	Vine	–	NE	0.83	56	JH-149
Caprifoliaceae	*Lonicera japonica* Thunb.	忍冬	Yin dong	Heat-clearing and detoxifying, promote blood circulation	Stem	Vine	–	NE	1.08	48	JH-085
Celastraceae	*Celastrus orbiculatus* Thunb.	南蛇藤	Nan nang mei	Heat-clearing and detoxifying, rheumatism	Fruit	Vine	–	NE	1.07	34	JH-287
Celastraceae	*Euonymus fortunei* (Turcz.) Hand.-Mazz.	扶芳藤	Fu fang mei	Relaxing tendons and activating collaterals	Stem, leaf	Shrub	–	NE	1.07	53	JH-066
Celastraceae	*Tripterygium wilfordii* Hook. f.	雷公藤	Bu ong mei	Rheumatism	Whole plant	Shrub	–	NE	0.80	48	JH-118
Chloranthaceae	*Chloranthus fortunei* (A. Gray) Solms-Laub.	丝穗金粟兰	Si sui jin su lan	Rheumatism, cold, heat-clearing and detoxifying, relieve cough	Whole plant	Herb	–	NE	1.39	73	JH-055
Colchicaceae	*Disporum cantoniense* (Lour.) Merr.	万寿竹	Wan shou lao	Relieve cough, promote digestion	Rhizome	Herb	–	NE	1.06	40	JH-214
Commelinaceae	*Murdannia keisak* (Hassk.) Hand.-Mazz.	疣草	You mi	Heat-clearing and detoxifying, induce diuresis	Whole plant	Herb	–	NE	1.05	34	JH-093
Compositae	*Achillea millefolium* L.	蓍	Shi	Rheumatism, gynaecopathia	Leaf, flower	Herb	Chinese PharmacopeiaI (2015) p350	NE	1.04	32	JH-016
Compositae	*Artemisia argyi* Levl. et Van.	艾	Ai	Gynaecopathia	Whole plant	Herb	–	NE	0.80	93	JH-005
Compositae	*Artemisia capillaris* Thunb.	茵陈蒿	Yin chen hao	Promote digestion, eliminating inflammation	Leaf	Herb	Japanese Pharmacopoeia 17	NE	1.02	35	JH-062
Compositae	*Artemisia dubia* Wall. ex Bess.	牛尾蒿	Ong dui hao	Rheumatism, heat-clearing and detoxifying, eliminating inflammation	Whole plant	Herb	Tibetan medicineIp16	NE	1.25	34	JH-156
Compositae	*Artemisia princeps* Pamp	魁蒿	Kui hao	Rheumatism, nourishing, gynaecopathia, eliminating inflammation, stop bleeding	Leaf	Herb	–	NE	1.74	9	JH-245
Compositae	*Aster tataricus* L. f.	紫菀	Zi wan	Heat-clearing and detoxifying	Root	Herb	Chinese PharmacopeiaI (2015) p342	NE	1.02	35	JH-003
Compositae	*Centipeda minima* (L.) A. Br. et Aschers.	石胡荽	Lao bie hu sui	Rheumatism, promote blood circulation, eliminating inflammation	Whole plant	Herb	–	NE	1.24	52	JH-162
Compositae	*Cirsium japonicum* Fisch. ex DC.	蓟	Ji	Nourishing, gynaecopathia, promote blood circulation, stop bleeding, eliminating inflammation	Whole plant, root	Herb	–	NE	1.68	26	JH-215
Compositae	*Dendranthema lavandulifolium* (Fisch. ex Trautv.) Ling & Shih	甘菊	Gan ju	Heat-clearing and detoxifying	Whole plant	Herb	–	NE	0.80	39	JH-166
Compositae	*Dendranthema morifolium* (Ramat.) Tzvel.	菊花	Ju ban	Heat-clearing and detoxifying, rheumatism, improve eyesight	Flower	Herb	Chinese PharmacopeiaI (2015) p310	NE	1.24	68	JH-047
Compositae	*Farfugium japonicum* (L. f.) Kitam.	大吴风草	Lu wu jia mi	Gynaecopathia, relieve cough	Root	Herb	–	NE	1.01	34	JH-280
Compositae	*Gerbera anandria* (L.) Sch.-Bip.	大丁草	Lu ding mi	Hepatitis	Whole plant	Herb	–	NE	0.80	43	JH-255
Compositae	*Gerbera piloselloides* (Linn.) Cass.	毛大丁草	Bie lu ding mi	Heat-clearing and detoxifying, eliminating inflammation, infantile malnutrition	Whole plant	Herb	–	NE	1.24	43	JH-223
Compositae	*Grangea maderaspatana* (L.) Poir.	田基黄	Lin ji yang	Heat-clearing and detoxifying	Whole plant	Herb	–	NE	0.79	71	JH-201
Compositae	*Gynura japonica* (Thunb.) Juel.	菊三七	Ju fang qie	Diabetes, infantile malnutrition	Whole plant	Herb	–	NE	1.01	46	JH-137
Compositae	*Inula japonica* Thunb.	旋覆花	Xuan fu ban	Infantile malnutrition	Root, leaf, flower	Herb	Chinese PharmacopeiaI (2015) p325	NE	0.79	15	JH-172
Compositae	*Kalimeris indica* (L.) Sch. -Bip.	马兰	Ma lan	Heat-clearing and detoxifying, relieve cough	Whole plant	Herb	–	NE	1.00	38	JH-188
Compositae	*Senecio scandens* Buch.-Ham. ex D. Don	千里光	Qian lei guang	Skin disease, improve eyesight, heat-clearing and detoxifying	Whole plant	Herb	Chinese PharmacopeiaI (2015) p33	NE	1.22	63	JH-076
Convolvulaceae	*Cuscuta chinensis* Lam.	菟丝子	Tu si zei	Nourishing	Seed	Herb	Chinese PharmacopeiaI (2015) p309	NE	0.78	21	JH-286
Convolvulaceae	*Dichondra repens* Forst.	马蹄金	Ma dei jin	Heat-clearing and detoxifying	Whole plant	Herb	–	NE	0.78	51	JH-270
Crassulaceae	*Sedum emarginatum* Migo	凹叶景天	Ao nong jing lu	Heat-clearing and detoxifying, stop bleeding, hepatitis	Whole plant	Herb	–	NE	1.21	67	JH-123
Crassulaceae	*Sedum kamtschaticum* Fisch.	堪察加景天	Kan cha jia jing lu	Eliminating inflammation, promote blood circulation, stop bleeding	Whole plant	Herb	–	NE	1.21	2	JH-242
Cruciferae	*Rorippa indica* (L.) Hiern.	蔊菜	Han cai	Stop bleeding, relieve cough	Whole plant	Herb	Chinese PharmacopeiaI (1977) p624	NE	1.00	43	JH-092
Cucurbitaceae	*Hemsleya macrosperma* C. Y. Wu ex C. Y. Wu et C. L. Chen	罗锅底	Luo ceng di	Heat-clearing and detoxifying, gastrointestinal disease	Tuber	Vine	–	NE	0.99	37	JH-283
Cucurbitaceae	*Thladiantha dubia* Bunge	赤瓟	Chi bo	Heat-clearing and detoxifying, promote blood circulation, relieve cough	Fruit, root	Shrub	–	NE	1.21	18	JH-187
Drynaria	*Pseudodrynaria coronans* (Wall. ex Mett.) Ching	崖姜	Ya su	Rheumatism, nourishing, relaxing tendons and activating collaterals	Rhizome	Fern	–	NE	1.37	63	JH-183
Equisetaceae	*Equisetum arvense* L.	问荆	Nai jin	Stop bleeding	Whole plant	Fern	–	NE	0.77	25	JH-289
Equisetaceae	*Equisetum ramosissimum* Desf. subsp. *debile* (Roxb. ex Vauch.) Hauke	笔管草	Ba gu mi	Improve eyesight, induce diuresis	Whole plant	Fern	–	NE	0.97	42	JH-197
Euphorbiaceae	*Glochidion puberum* (L.) Hutch.	算盘子	Fu bian zei	Heat-clearing and detoxifying, promote digestion, promote blood circulation	Root	Shrub	–	NE	1.20	39	JH-091
Fabaceae	*Callerya speciosa* (Champ. ex Benth.) Schot	美丽鸡血藤	Hao zui jia jiang mei	Nourishing, heat-clearing and detoxifying, relaxing tendons and activating collaterals	Root	Vine	–	NE	1.37	38	JH-269
Fabaceae	*Desmodium multiflorum* DC.	饿蚂蝗	E ma huang	Heat-clearing and detoxifying, infantile malnutrition	Flower, branch	Shrub	–	NE	0.97	36	JH-144
Fabaceae	*Entada phaseoloides* (Linn.) Merr.	榼藤	Ke mei	Rheumatism, nourishing, promote blood circulation	Stem	Vine	–	NE	1.20	15	JH-143
Fabaceae	*Flemingia philippinensis* Merr. et Rolfe	千斤拔	Qin jiang ben	Nourishing	Root	Shrub	–	NE	0.77	51	JH-012
Fabaceae	*Gleditsia sinensis* Lam.	皂荚	Zao jia	Eliminate phlegm, induce diuresis	Pod, seed, shoot thorn	Tree	–	NE	0.95	42	JH-256
Fabaceae	*Indigofera decora* Lindl. var. *ichangensis* (Craib) Y. Y. Fang et C. Z. Zheng	宜昌木蓝	Yi chang mu lan	High fever	Root	Shrub	–	NE	0.77	42	JH-080
Fabaceae	*Kummerowia striata* (Thunb.) Schindl.	鸡眼草	Jia mu jin mi	Heat-clearing and detoxifying, promote blood circulation, promote digestion	Whole plant	Herb	–	NE	1.19	67	JH-290
Fabaceae	*Lespedeza cuneata* G. Don	截叶铁扫帚	Jie nong li bu. gan dao	Heat-clearing and detoxifying, improve eyesight, infantile malnutrition	Whole plant	Shrub	–	NE	1.19	18	JH-292
Gramineae	*Lophatherum gracile* Brongn.	淡竹叶	Cuan lao nong	Heat-clearing and detoxifying, relieve cough, induce diuresis	Root	Herb	Chinese PharmacopeiaI (2015) p328	NE	1.19	72	JH-243
Gramineae	*Pennisetum alopecuroides* (L.) Spreng.	狼尾草	Lang dui mi	Heat-clearing and detoxifying, relieve cough	Whole plant	Herb	–	NE	0.95	28	JH-106
Gramineae	*Saccharum spontaneum* L.	甜根子草	Gan mi zei mi	Heat-clearing and detoxifying, cold, relieve cough	Rhizome, stem	Herb	–	NE	1.18	57	JH-276
Guttiferae	*Hypericum japonicum* Thunb. ex Murray	地耳草	Dao mu nong mi	Heat-clearing and detoxifying, promote blood circulation, promote digestion	Whole plant	Herb	Chinese PharmacopeiaI (1977) p198	NE	1.18	42	JH-189
Guttiferae	*Hypericum monogynum* L.	金丝桃	Jin si tao	Rheumatism, relieve cough, stomachache	Root	Shrub	–	NE	1.18	48	JH-140
Guttiferae	*Hypericum sampsonii* Hance	元宝草	Yuan bao mi	Gynaecopathia, heat-clearing and detoxifying, relaxing tendons and activating collaterals	Whole plant	Herb	Chinese PharmacopeiaI (1977) p79	NE	1.37	52	JH-131
Juncaceae	*Juncus effusus* L.	灯心草	Dang fin mi	Heat-clearing and detoxifying, induce diuresis, respiratory disease, relieve cough	Spith	Herb	Chinese PharmacopeiaI (2015) p147	NE	1.36	60	JH-262
Labiatae	*Leonurus artemisia* (Laur.) S. Y. Hu	益母草	Yi mu cao	Heat-clearing and detoxifying	Whole plant	Herb	Chinese PharmacopeiaI (2015) p290	NE	0.77	67	JH-075
Labiatae	*Lycopus lucidus* Turcz.	地笋	Dao bia	Rheumatism	Whole plant	Herb	–	NE	0.76	38	JH-033
Labiatae	*Mosla chinensis* Maxim.	石香薷	Shi xiang ru	Heatstroke	Whole plant	Herb	–	NE	0.76	9	JH-019
Labiatae	*Pogostemon auricularius* (L.) Kassk.	珍珠菜	Zhen zhu lai	Heat-clearing and detoxifying	Whole plant	Herb	–	NE	0.74	40	JH-239
Labiatae	*Prunella vulgaris* L.	夏枯草	Xia ku cao	Improve eyesight, promote blood circulation	Fruit cluster, flower	Herb	Chinese PharmacopeiaI (2015) p280	NE	0.74	67	JH-179
Labiatae	*Scutellaria barbata* D. Don	半枝莲	Dan zhi lian	Heat-clearing and detoxifying, induce diuresis, cold	Whole plant	Herb	Chinese PharmacopeiaI (2015) p118	NE	1.18	36	JH-042
Lauraceae	*Cinnamomum appelianum* Schewe	毛桂	Mao gui	Rheumatism	Bark, root	Tree	–	NE	0.73	42	JH-088
Liliaceae	*Anemarrhena asphodeloides* Bunge	知母	Zei ma	Promote digestion, gynaecopathia	Rhizome	Herb	Chinese PharmacopeiaI (2015) p212	NE	0.94	13	JH-113
Liliaceae	*Aspidistra elatior* Blume	蜘蛛抱蛋	Geng you luo jiao	Nourishing, promote blood circulation, relieve cough	Rhizome	Herb	–	NE	0.94	62	JH-174
Liliaceae	*Aspidistra retusa* K. Y. Lang et S. Z. Huang	广西蜘蛛抱蛋	Jiang fai geng you luo jiao	Nourishing, promote blood circulation, relieve cough	Rhizome	Herb	–	NE	0.93	37	JH-130
Liliaceae	*Liriope platyphylla* Wang et Tang	阔叶山麦冬	Jiag nong geng me dong	Nourishing	Tuber	Herb	–	NE	0.73	59	JH-271
Liliaceae	*Ophiopogon bodinieri* Levl.	沿阶草	Yan gai mi	Heat-clearing and detoxifying	Tuber	Herb	–	NE	0.72	57	JH-069
Liliaceae	*Ophiopogon japonicus* (L. f.) Ker-Gawl.	麦冬	Me dong	Nourishing	Tuber	Herb	Chinese PharmacopeiaI (2015) p155	NE	0.72	61	JH-217
Liliaceae	*Polygonatum sibiricum* Delar. ex Redoute	黄精	Yang jing	Nourishing	Rhizome	Herb	Chinese PharmacopeiaI (2015) p306	NE	0.71	59	JH-236
Liliaceae	*Reineckia carnea* (Andr.) Kunth	吉祥草	Ji xiang mi	Heat-clearing and detoxifying, relieve cough	Whole plant	Herb	–	NE	0.93	59	JH-251
Loranthaceae	*Viscum articulatum* Burm. f.	扁枝槲寄生	Bian zhi hu ji sheng	Rheumatism, respiratory disease, promote blood circulation	Branch, leaf	Shrub	–	NE	1.18	49	JH-211
Loranthaceae	*Viscum diospyrosicolum* Hayata	棱枝槲寄生	Shi ji sheng	Rheumatism, heat-clearing and detoxifying, eliminating inflammation, relaxing tendons and activating collaterals	Whole plant	Phytoparasite	–	NE	1.60	43	JH-111
Loranthaceae	*Viscum liquidambaricolum* Hayata	枫香槲寄生	Feng xiang hu ji sheng	Rheumatism, relaxing tendons and activating collaterals, promote blood circulation, resolve phlegm to relieve cough	Branch, leaf	Phytoparasite	–	NE	1.60	37	JH-107
Lycopodoaceae	*Diphasiastrum complanatum* (L.) Holub	扁枝石松	Bian zhi shi song	Rheumatism	Whole plant	Herb	–	NE	0.68	65	JH-297
Lygodiaceae	*Lygodium japonicum* (Thunb.) Sw.	海金沙	Hai jin sha	Induce diuresis, calculus, rheumatism	Spore, whole plant	Herb	Chinese PharmacopeiaI (2015) p294	NE	1.17	59	JH-216
Lythraceae	*Lythrum salicaria* L.	千屈菜	Qin qu lai	Infantile malnutrition, stop bleeding	Whole plant	Herb	–	NE	0.93	13	JH-148
Lythraceae	*Rotala rotundifolia* (Buch.-Ham. ex Roxb.) Koehne	圆叶节节菜	Jun nong a a lai	Heat-clearing and detoxifying	Whole plant	Herb	–	NE	0.67	33	JH-272
Melastomataceae	*Melastoma dodecandrum* Lour.	地菍	Dao nian	Promote digestion	Whole plant	Shrub	–	NE	0.67	71	JH-263
Melastomataceae	*Memecylon scutellatum* (Lour.) Hook. et Arn.	细叶谷木	Fai nong cu diang	Heart disease	Flower	Shrub	–	NE	0.67	4	JH-157
Melastomataceae	*Osbeckia opipara* C. Y. Wu et C. Chen	朝天罐	Chao lu guan	Eliminating inflammation, promote digestion, heat-clearing and detoxifying, stop bleeding	Whole plant, root	Shrub	Chinese PharmacopeiaI (1977) p574	NE	1.34	42	JH-115
Menispermaceae	*Stephania cepharantha* Hayata	金线吊乌龟	Jin sui di wu gui	Eliminating inflammation	Tuber	Vine	–	NE	0.66	39	JH-168
Menispermaceae	*Stephania lincangensis* Lo et M. Yang	临沧地不容	Lin cang dao en rong	Heat-clearing and detoxifying, promote blood circulation, relieve pain	Tuber	Vine	–	NE	1.17	3	JH-053
Menispermaceae	*Tinospora sagittata* (Oliv.) Gagnep.	青牛胆	Men ong dan	Heat-clearing and detoxifying, eliminating inflammation, relieve pain	Tuber	Vine	–	NE	1.16	46	JH-231
Moraceae	*Ficus pumila* Linn.	薜荔	Xue li	Nourishing, rheumatism	Fruit	Shrub	–	NE	0.92	40	JH-002
Musaceae	*Musa basjoo* Sieb. & Zucc.	芭蕉	Ba jiao	Heart disease	Flower	Herb	–	NE	0.65	26	JH-006
Myrsinaceae	*Ardisia affinis* Hemsl.	细罗伞	Fai luo fan	Promote blood circulation	Root	Shrub	–	NE	0.63	56	JH-095
Myrsinaceae	*Ardisia chinensis* Benth.	小紫金牛	Fai zi jin ong	Promote blood circulation, heat-clearing and detoxifying, eliminating inflammation, stop bleeding	Whole plant	Shrub	–	NE	1.34	59	JH-001
Myrsinaceae	*Ardisia crenata* Sims var. *bicolor* (Walker) C. Y. Wu et C. Chen	朱砂根	Zhu sha jiang	Rheumatism, respiratory disease	Whole plant	Shrub	Chinese PharmacopeiaI (2015) p138	NE	0.92	53	JH-254
Myrsinaceae	*Ardisia japonica* (Thunb) Blume	紫金牛	Zi jin ong	Rheumatism, promote blood circulation, cold, relieve cough	Whole plant, root	Shrub	–	NE	1.33	62	JH-121
Myrsinaceae	*Embelia rudis* Hand.-Mazz.	网脉酸藤子	Wang me sui mei	Rheumatism	Whole plant	Shrub	–	NE	0.62	38	JH-004
Orchidaceae	*Bulbophyllum odoratissimum* (J. E. Smith) Lindl.	密花石豆兰	Mi ban lao bie de lan	Respiratory disease, infantile malnutrition, relax tendons and activate collaterals, eliminating inflammation	Whole plant	Herb	–	LC	1.57	41	JH-264
Orchidaceae	*Bulbophyllum pectinatum* Finet	长足石豆兰	Zao da lao bie de lan	Respiratory disease, relieve cough	Whole plant	Herb	–	VU	0.91	41	JH-041
Orchidaceae	*Dendrobium nobile* Lindl.	石斛	Lao bie hu	Diabetes, improve eyesight, nourishing, promote digestion	Stem	Herb	Chinese PharmacopeiaI (2015) p92	VU	1.33	66	JH-101
Orchidaceae	*Dendrobium officinale* Kimura et Migo	铁皮石斛	Li lao bie hu	Stomachache	Stem	Herb	Chinese PharmacopeiaI (2015) p282		0.61	41	JH-265
Orchidaceae	*Galeola lindleyana* (Hook. f. et Thoms.) Rchb. f.	毛萼山珊瑚	Mao e shan shan hu	Rheumatism, headache	Whole plant	Shrub	–	LC	0.91	52	JH-058
Orchidaceae	*Luisia morsei* Rolfe	钗子股	Chai zi gu	Rheumatism, respiratory disease, cold, cancer	Whole plant	Herb	–	LC	1.33	13	JH-133
Orchidaceae	*Spiranthes sinensis* (Pers.) Ames	绶草	Shou mi	Nourishing, heat-clearing and detoxifying	Whole plant	Herb	–	LC	1.16	11	JH-122
Papaveraceae	*Eomecon chionantha* Hance	血水草	Jiang wen mi	Promote blood circulation	Root, rhizome	Herb	–	NE	0.59	14	JH-219
Phyllanthaceae	*Phyllanthus urinaria* L.	叶下珠	Nong di zhu	Improve eyesight, heat-clearing and detoxifying, promote digestion	Whole plant, root	Herb	–	NE	1.15	69	JH-083
Pipperaceae	*Piper betle* L.	蒌叶	Lou nong	Heat-clearing and detoxifying, eliminating inflammation, cold	Stem, leaf	Vine	–	NE	1.15	40	JH-029
Pittosporaceae	*Pittosporum glabratum* Lindl.	光叶海桐	Jiang nong hai tong	Tuberculosis	Seed, bark	Shrub	–	NE	0.58	40	JH-173
Polygalaceae	*Polygala japonica* Houtt.	瓜子金	Jin gua zei	Eliminating phlegm, heat-clearing and detoxifying	Whole plant	Herb	Chinese PharmacopeiaI (2015) p112	NE	0.91	38	JH-037
Polygalaceae	*Polygala tenuifolia* Willd.	远志	Gu zei	Nourishing, eliminating phlegm, strengthen muscles and bones	Bark	Herb	Chinese PharmacopeiaI (2015) p156	NE	1.14	39	JH-191
Polygonaceae	*Fagopyrum dibotrys* (D. Don) Hara	金荞麦	Jin qiao me	Heat-clearing and detoxifying, promote blood circulation, calculus	Root, rhizome	Herb	Chinese PharmacopeiaI (2015) p218	LC	1.14	52	JH-230
Polygonaceae	*Fallopia multiflora* (Thunb.) Harald.	何首乌	Huo shou wu	Nourishing	Tuber	Herb	Chinese PharmacopeiaI (2015) p175	NE	0.52	55	JH-192
Polypodiaceae	*Lepidogrammitis drymoglossoides* (Baker) Ching	抱石莲	Luo lao bie lian	Heat-clearing and detoxifying, induce diuresis, stop bleeding	Whole plant	Fern	–	NE	1.13	54	JH-057
Polypodiaceae	*Lepisorus thunbergianus* (Kaulf.) Ching	瓦韦	Wa wei	Heat-clearing and detoxifying, induce diuresis, relieve cough	Whole plant	Fern	–	NE	1.13	52	JH-116
Polypodiaceae	*Microsorum fortunei* (T. Moore) Ching	江南星蕨	Jiang nan xing jue	Rheumatism	Whole plant	Fern	–	NE	0.52	62	JH-059
Portulacaceae	*Portulaca oleracea* L.	马齿苋	Ma chi xian	Heat-clearing and detoxifying, eliminating phlegm	Whole plant	Herb	Chinese PharmacopeiaI (2015) p49	NE	0.91	37	JH-007
Primulaceae	*Plantago asiatica* L.	车前	Qi dan men	Heat-clearing and detoxifying, induce diuresis, eliminating phlegm	Whole plant	Herb	–	NE	1.13	69	JH-018
Ranunculaceae	*Clematis henryi* Oliv.	单叶铁线莲	Dan nong li sui lian	Eliminating phlegm, relieve pain, relieve cough	Root, leaf	Vine	--, c	NE	1.13	1	JH-026
Ranunculaceae	*Clematis uncinata* Champ.	柱果铁线莲	Zhu guo li sui lian	Rheumatism, stop bleeding, toothache, relaxing tendons and activating collaterals	Root, leaf	Vine	–	NE	1.32	1	JH-155
Rhamnaceae	*Rhamnus crenata* Sieb. et Zucc.	长叶冻绿	Nong da dong lu	Heat-clearing and detoxifying	Whole plant	Shrub	–	NE	0.48	2	JH-071
Rhamnaceae	*Rhamnus globosa* Bunge	圆叶鼠李	Jun nong na jun li	Heat-clearing and detoxifying	Fruit	Shrub	–	NE	0.47	9	JH-273
Rhamnaceae	*Sageretia thea* (Osbeck) Johnst.	雀梅藤	Que mei mei	Eliminating phlegm, rheumatism	Aerial part	Shrub	–	NE	0.47	41	JH-198
Rosaceae	*Geum aleppicum* Jacq.	路边青	Jiao leng men	Rheumatism, heat-clearing and detoxifying, relieve pain	Whole plant	Herb	–	NE	1.13	60	JH-100
Rosaceae	*Potentilla discolor* Bge.	翻白草	Bian bei mi	Heat-clearing and detoxifying, stop bleeding, diabetes	Whole plant	Herb	Chinese PharmacopeiaI (2015) p383	NE	1.12	45	JH-190
Rosaceae	*Sanguisorba officinalis* L.	地榆	Di yu	Heat-clearing and detoxifying, stop bleeding, relieve pain	Root	Herb	Chinese PharmacopeiaI (2015) p126	NE	1.11	35	JH-209
Rubiaceae	*Damnacanthus indicus* Gaertn.	虎刺	Hu ci	Infantile malnutrition, nourishing, relieve pain, cold, hepatitis	Whole plant	Shrub	Chinese PharmacopeiaI (1977) p341	NE	1.29	72	JH-234
Rubiaceae	*Hedyotis auricularia* L.	耳草	Tu nong mi	Heat-clearing and detoxifying, promote digestion, relieve cough, cold, promote blood circulation	Leaf	Herb	–	NE	1.54	62	JH-206
Rubiaceae	*Paederia scandens* (Lour.) Merr.	鸡矢藤	Jia gai mei	Rheumatism, promote digest, heat-clearing and detoxifying	Whole plant	Vine	Chinese PharmacopeiaI (1977) p312	NE	1.11	31	JH-074
Rubiaceae	*Serissa serissoides* (DC.) Druce	白马骨	Bei ma mei	Rheumatism, heat-clearing and detoxifying, relax tendons and activate collaterals	Whole plant	Shrub	–	NE	1.11	60	JH-051
Saururaceae	*Houttuynia cordata* Thunb	蕺菜	Ji lai	Heat-clearing and detoxifying, respiratory disease, heatstroke	Root, leaf	Herb	–	NE	1.11	9	JH-089
Saururaceae	*Saururus chinensis* (Lour.) Baill.	三白草	Bu bei mi	Gynaecopathia	Whole plant	Herb	Chinese PharmacopeiaI (2015) p12	NE	0.46	65	JH-061
Saxifragaceae	*Astilbe rivularis* Buch.-Ham. ex D. Don	溪畔落新妇	Xi pan luo xin fu	Rheumatism, promote blood circulation, relieve pain, promote digestion	Rhizome	Herb	–	NE	1.29	16	JH-032
Stachyuraceae	*Stachyurus chinensis* Franch.	中国旌节花	Zhong guo sheng jie hua	Gynaecopathia, heat-clearing and detoxifying, eliminating inflammation, induce diuresis	Pith	Shrub	–	NE	1.29	35	JH-068
Stemonaceae	*Stemona tuberosa* Lour.	大百部	Dong bei bo	Respiratory disease	Tuber	Vine	–	NE	0.45	78	JH-281
Sterculiaceae	*Pterospermum heterophyllum* Hance	翻白叶树	Bian bei nong diang	Rheumatism, relax tendons and activate collaterals, relieve pain	Whole plant	Tree	–	NE	1.11	39	JH-109
Taccaceae	*Schizocapsa plantaginea* Hance	裂果薯	Nong hu duai	Heat-clearing and detoxifying, eliminating inflammation, stop bleeding	Rhizome	Herb	–	NE	1.11	54	JH-011
Trilliaceae	*Paris polyphylla* Sm.	七叶一枝花	Qi ye yi zhi hua	Heat-clearing and detoxifying, relieve cough	Rhizome	Herb	–		0.91	30	JH-260
Umbelliferae	*Bupleurum chinense* DC.	北柴胡	Bei chai hu	Eliminating inflammation, heat-clearing and detoxifying, cold, fever	Root	Herb	–	NE	1.28	31	JH-030
Umbelliferae	*Hydrocotyle sibthorpioides* Lam.	天胡荽	Tian hu sui	Heat-clearing and detoxifying, promote digest, infantile malnutrition	Whole plant	Herb	–	NE	1.10	69	JH-060
Umbelliferae	*Peucedanum guangxiense* Shan et Sheh	广西前胡	Jiang fai qian hu	Cold, rheumatism	Root	Herb	–	NE	0.90	36	JH-024
Umbelliferae	*Sanicula chinensis* Bunge	变豆菜	Ben de lai	Relieve cough, promote digestion, heat-clearing and detoxifying, eliminating inflammation	Whole plant	Herb	–	NE	1.28	19	JH-025
Urticaceae	*Boehmeria nivea* (L.) Gaudich.	苎麻	Zhu ma	Heat-clearing and detoxifying, induce diuresis, stop bleeding, nourishing	Rhizome, leaf	Shrub	–	NE	1.26	33	JH-291
Urticaceae	*Parietaria micrantha* Ledeb.	墙草	Jiong mi	Heat-clearing and detoxifying, promote digestion	Whole plant	Herb	–	NE	0.90	9	JH-099
Urticaceae	*Pilea cavaleriei* Levl.	波缘冷水花	Bo yuan wen nan ban	Relieve cough, heat-clearing and detoxifying	Whole plant	Herb	–	NE	0.90	41	JH-194
Urticaceae	*Pilea cavaleriei* Levl. subsp. *valida* C. J. Chen	石油菜	Lao bie you lai	Heat-clearing and detoxifying, relieve pain	Whole plant	Herb	–	NE	0.90	37	JH-063
Usneaceae	*Usnea diffracta* (Vain.) Articus	松萝	Song luo	Rheumatism	Whole plant	Thallus	Uygur medicine p49	NE	0.33	71	JH-147
Verbenaceae	*Clerodendrum philippinum* Schauer var. *simplex* Moldenke	臭茉莉	Zui mo li	Rheumatism, promote blood circulation, relieve pain, heat-clearing and detoxifying, promote digestion	Root, leaf, whole plant	Shrub	–	NE	1.52	46	JH-164
Verbenaceae	*Clerodendrum cyrtophyllum* Turcz.	大青	Dong qin	Heat-clearing and detoxifying, rheumatism	Root, leaf	Shrub	–	NE	0.89	51	JH-142
Verbenaceae	*Verbena officinalis* L.	马鞭草	Ma bian mi	Rheumatism, heat-clearing and detoxifying, promote blood circulation, eliminating inflammation	Whole plant	Herb	Chinese PharmacopeiaI (2015) p52	NE	1.25	45	JH-135
Violaceae	*Viola inconspicua* Blume	长萼堇菜	Da e jin lai	Heat-clearing and detoxifying, promote blood circulation	Whole plant	Herb	–	NE	0.89	10	JH-252
Vitaceae	*Ampelopsis grossedentata* (Hand.-Mazz.) W. T. Wang	显齿蛇葡萄	Xian chi nan pu tao	Respiratory disease, heat-clearing and detoxifying, hypertension	Stem, leaf	Vine	–	NE	1.10	46	JH-120
Vitaceae	*Cayratia japonica* (Thunb.) Gagnep.	乌蔹莓	Wu lian mei	Heat-clearing and detoxifying, induce diuresis	Whole plant	Vine	–	NE	0.88	39	JH-108
Vitaceae	*Euphorbia humifusa* Willd. ex Schlecht.	地锦	Dao jin	Rheumatism, promote blood circulation	Root, stem, fruit	Vine	–	NE	0.88	9	JH-266
Xanthorrhoeaceae	*Hemerocallis citrina* Baroni	黄花菜	Yang ban lai	Heat-clearing and detoxifying, nourishing	Root, flower	Herb	–	NE	0.87	19	JH-090

*VU* vulnerable, *LC* least concern, *NE* not evaluated

### Statistical analysis

Cognitive salience (CS) [8] and use value (UV) [9] were applied to determine the greatest cognitive and cultural importance of these medical plants in Jianghua.

Free-listing is a method to obtain cognitive salience from relatively large samples [10, 11]. Interviewers collected traditional knowledge from large samples of free-lists which reveal cognitive salience from individuals’ local knowledge. The measure of cognitive salience includes both list position and list frequency irrespective of list length or number of respondents [8, 12]. We interviewed 215 informants and recorded 215 free-lists; here, we calculated the mean cognitive salience (CS) for each listed species,

$$ \mathrm{CS}=\frac{\left[\sum B+F-1\right]}{\left[2Z-1\right]} $$

$$ B=\frac{\left[K-r(i)\right]}{\left[K-1\right]} $$

*F* is the number of lists where the particular species is mentioned in all lists while *Z* is the number of informants. *B* determines how one plant precedes other plants mentioned in a respondent’s list. *K* is the number of listed species in one informant, and *r* (*i*) is the *i*th order of each plant’s list position.

The closer to the first position (or rank) the item(s) are, the greater the cognitive salience of item(s) is deemed to be.

The use value (UV) is to quantitatively evaluate the relative importance of species [13–15] used by Yao people,

$$ UV=\sum Ui/N $$

where *Ui* refers to the number of medical use cited by an informant for per species and *N* is the total number of all informants. When there are many use reports mentioned for one plant, it indicates the use value of this plant is high.

The coefficient of similarity (*S*) of herbal tea plants between Jianghua and Lingnan regions was calculated by the following formula: *S* = 2*c*/(*a* + *b*) (*a* and *b* are species used by Jianghua and Lingnan regions, respectively; *c* are species in common use) [16].

Chi-square analysis was applied to find whether the traditional knowledge of herbal tea such as plant life form and plant part(s) used varied considerably between Jianghua and Lingnan.

## Results

### Medicinal plant species sold for herbal tea at the traditional medicinal markets

#### Plant species and life form

**Fig. 2:**
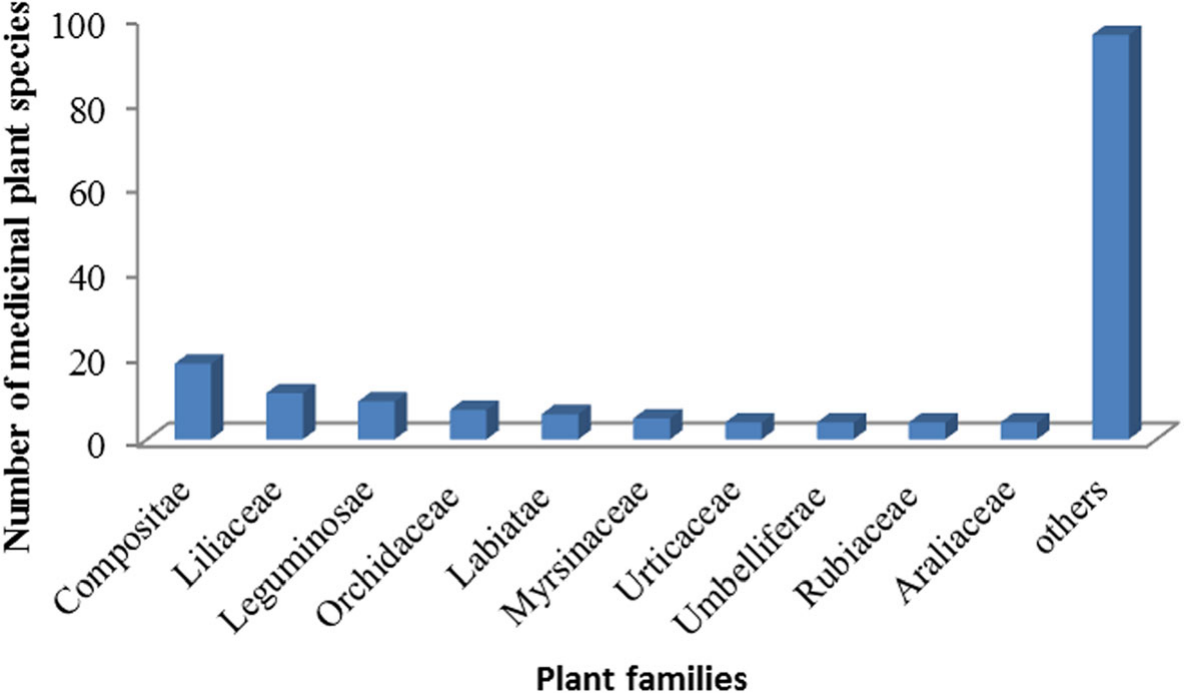
Dominant medicinal plant families used for herbal tea in the Jianghua traditional medicinal market, China, where *f* > 3, and f is the number of species in a family; for families where *f* < 3, these were summarized as “others”

**Fig. 3:**
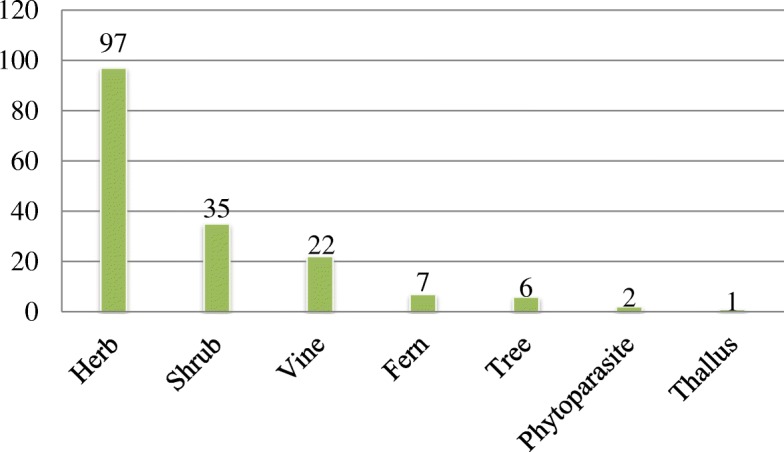
Habitat of herbs used for herbal tea in Jianghua

According to the results of the taxonomical identification, the medicinal plants used for herbal tea belong to 169 species, grouped into 142 genera and 66 families. In alphabetical order of the family, they are presented in Table 1. Further analyses on the plant families show that Compositae has 18 species, making it the dominant family. Liliaceae, Leguminosae, Orchidaceae, Labiatae, and Myrsinaceae are represented by 11, 9, 7, 6, and 5 species, followed by Urticaceae, Umbelliferae, Rubiaceae, and Araliaceae, with 4 species each, and 13 families containing 3 species, 14 families containing 2 species, and 29 families containing 1 species (Fig. 2). Of the 169 species, the most frequent habits of medicinal plants were herbs (97 spp.), followed by shrubs (35 spp.), vines (22 spp.), ferns (7 spp.), trees (6 spp.), phytoparasites (2 spp.), and thalli (1 sp.) (Fig. 3).

#### Part(s) used

**Fig. 4:**
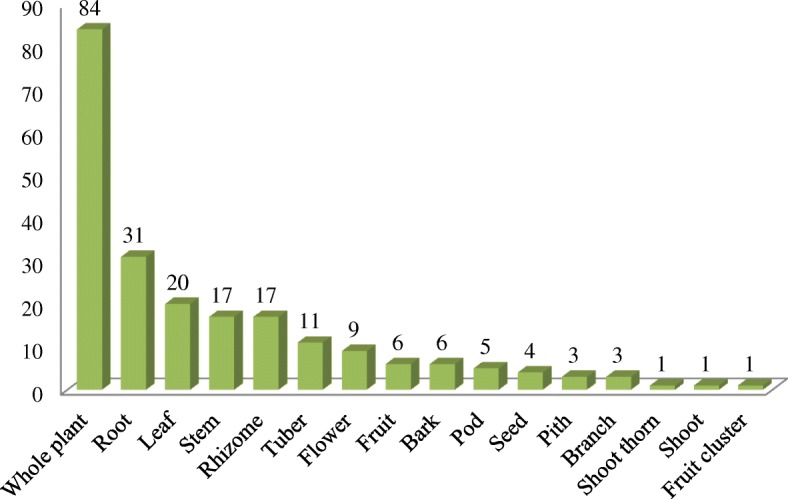
Plant parts used for herbal tea in Jianghua

In this study, the analysis revealed that there were 16 kinds of plant parts that were used for herbal tea as medicinal materials. The whole plant was the most commonly used plant part (38.4%), followed by root (14.2%), leaf (9.13%), stem (7.76%), rhizome (7.76%), and tuber (5.02%) (Fig. 4). The study also found that some other plant parts, such as the flower, fruit, bark, pod, seed, pith, branch, shoot thorn, shoot, and fruit cluster, are used less frequently.

#### Conservation status

According to the evaluation criteria established by the International Union for Conservation of Nature (http://rep.iplant.cn/protlist), three of these species are listed on “China’s red list” and registered as vulnerable (VU), which means that they are at the highest risk for endangerment, namely, *Dysosma versipellis*, *Bulbophyllum pectinatum*, and *Dendrobium nobile*. In addition, five species are categorized under least concern (LC), which is a lower category of risk; they are *Bulbophyllum odoratissimum*, *Galeola lindleyana*, *Luisia morsei*, *Spiranthes sinensis*, and *Fagopyrum dibotrys*, and 159 species were not evaluated (NE) while *Paris polyphylla* is listed as second degree national protective plants and *Dendrobium officinale* is listed as first degree national protective plants. There is a need to investigate and provide proper management to avoid a shortage.

#### Medicinal uses

**Fig. 5:**
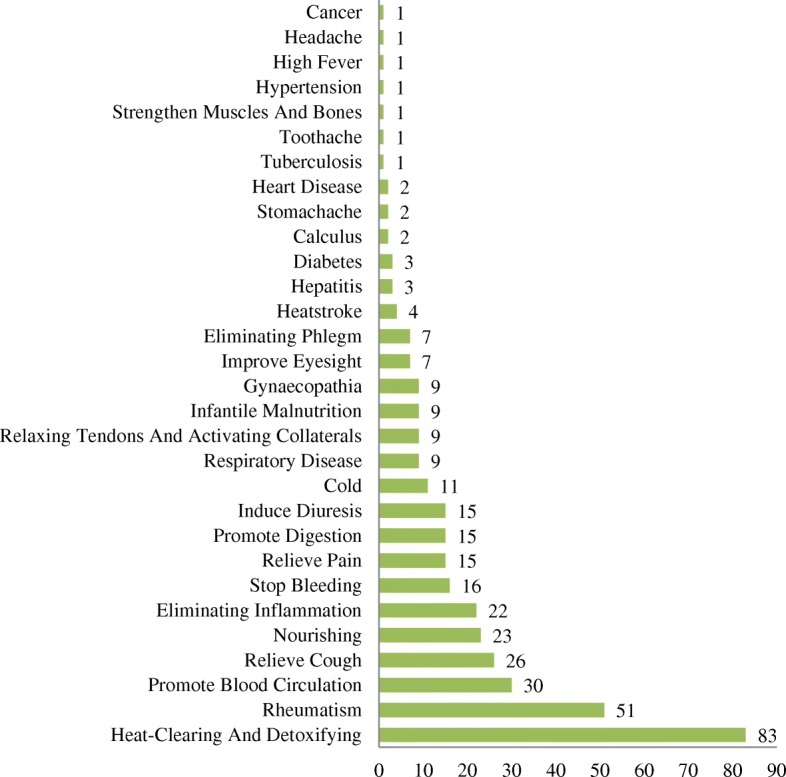
Plant species involved in each medical function

In our study, a total of 30 medicinal uses were recorded, and heat-clearing and detoxifying was the most common medicinal function, followed by treating rheumatism and promoting blood circulation (Fig. 5). In Jianghua, 49.11% of the medicinal plant species (83 spp.) are used for heat-clearing and detoxifying, 30.18% for treating rheumatism, 17.75% for promoting blood circulation, and 15.38% for relieving cough.

### Cultural and medical significance of species

The cognitive salience of 169 species ranked from 0.012 to 0.343 (Table 1); 10 species listed as the most salient are *Artemisia argyi* Levl. et Van., *Stemona tuberosa* Lour., *Chloranthus fortunei* (A. Gray) Solms-Laub., *Grangea maderaspatana* (L.) Poir., *Lophatherum gracile* Brongn., *Usnea diffracta* (Vain.) Articus, *Melastoma dodecandrum* Lour., *Damnacanthus indicus* Gaertn., *Plantago asiatica* L., and *Leonurus artemisia* (Laur.) S. Y. Hu. The most 20 salient species are listed in Table 2. The greater the value of cognitive salience, the more culturally important the species is. For example, the highest value refers to *Artemisia argyi* Levl. et Van., which is a fundamental medicinal plant to local people. The least value of cognitive salience is *Clematis uncinata* Champ.

**Table 2:** Cognitive salience for 20 most value species

Scientific name	Chinese name	Cognitive salience
*Artemisia argyi* Levl. et Van.	艾	0.343
*Stemona tuberosa* Lour.	大百部	0.278
*Chloranthus fortunei* (A. Gray) Solms-Laub.	丝穗金粟兰	0.264
*Grangea maderaspatana* (L.) Poir.	田基黄	0.261
*Lophatherum gracile* Brongn.	淡竹叶	0.251
*Usnea diffracta* (Vain.) Articus	松萝	0.251
*Melastoma dodecandrum* Lour.	地菍	0.249
*Damnacanthus indicus* Gaertn.	虎刺	0.247
*Plantago asiatica* L.	车前	0.242
*Leonurus artemisia* (Laur.) S. Y. Hu	益母草	0.241
*Prunella vulgaris* L.	夏枯草	0.239
*Kummerowia striata* (Thunb.) Schindl.	鸡眼草	0.238
*Hydrocotyle sibthorpioides* Lam.	天胡荽	0.237
*Dendranthema morifolium* (Ramat.) Tzvel.	菊花	0.237
*Sedum emarginatum* Migo	凹叶景天	0.236
*Phyllanthus urinaria* L.	叶下珠	0.233
*Dendrobium nobile* Lindl.	石斛	0.230
*Saururus chinensis* (Lour.) Baill.	三白草	0.224
*Diphasiastrum complanatum* (L.) Holub	扁枝石松	0.224
*Acorus tatarinowii* Schott	石菖蒲	0.223

The use value of 169 species ranked from 0.33 to 1.74. They are *Artemisia princeps* (1.74), *Viscum liquidambaricola* (1.68), *Viscum diospyrosicola* (1.60), *Hedyotis auricularia* (1.60), *Clerodendrum chinense* var. *simplex* (1.57), *Cirsium japonicum* (1.54), *Achyranthes aspera L.* (1.52), *Schefflera octophylla* (Linn.) Frodin (1.51), *Panax japonicus* (T. Nees) C. A. Mey. (1.48), and *Pseudodrynaria coronans* (1.47), which are widely and frequently used by local people.

#### Comparison of medicinal plant tradition in Jianghua and Lingnan

A comparison of plant materials commonly used for herbal tea in Jianghua and Lingnan shows that there are 23 plant species in total used for herbal tea (Table 3), and Compositae is the predominant family in two regions. For part(s) used for herbal tea, no matter whether they are from Lingnan or Jianghua, the vendors like to use whole plants and roots to prepare herbal tea. By comparing, we found that the common functions of the herbal tea produced by the people both in Jianghua and Lingnan are heat-clearing, detoxifying, and treating rheumatism.

**Table 3:** A comparison of plant materials commonly used for herbal tea in Jianghua and Lingnan

	Plant species	Jianghua		Lingnan	
Consistency in efficacy	*Achyranthes aspera*	Heat-clearing and detoxifying, rheumatism, nourishing, relieve pain	Whole plant	Clear heat and purge fire	Root
	*Achyranthes bidentata*	Nourishing	Root	Activate blood and remove blood stasis, nourish the liver and the kidney, strengthen bones and muscles, alleviate edema and relieve stranguria, conduct blood-fire to go downward	Root
	*Mahonia fortunei*	Heat-clearing and detoxifying	Root, stem	Nourish yin and clear heat, warm lung and stop cough	Leaf
	*Lonicera confusa*	Heat-clearing and detoxifying	Flower, stem, leaf	Clear heat and relieve toxicity, disperse wind	Flower, stem
	*Lonicera japonica*	Heat-clearing and detoxifying, promote blood circulation	Stem	Clear heat and relieve toxicity, disperse wind	Flower, stem
	*Cirsium japonicum*	Nourishing, gynaecopathia, promote blood circulation, stop bleeding, eliminating inflammation	Whole plant, root	Cool the blood and stop bleeding, eliminate toxic materials to treat carbuncle	Aerial part, root
	*Dendranthema morifolium*	Heat-clearing and detoxifying, rheumatism, improve eyesight	Flower	Clear heat and relieve toxicity	Flower
	*Lophatherum gracile*	Heat-clearing and detoxifying, relieve cough, induce diuresis	Root	Clear heat, sedative	Leaf, root
	*Juncus effusus*	Heat-clearing and detoxifying, induce diuresis, respiratory disease, relieve cough	Spith	Clear away liver-heat and lower the fire	Pith
	*Prunella vulgaris*	Improve eyesight, promote blood circulation	Fruit cluster, flower	Clear liver, purge fire, resolve knots, dissolve swelling, pacify liver and improve eyesight.	Whole plant
	*Gleditsia sinensis*	Eliminate phlegm, induce diuresis	Pod, seed, shoot thorn	Relieve pathological heat and remove dampness through diuresis	Fruit
	*Anemarrhena asphodeloides*	Promote digestion, gynaecopathia	Rhizome	Strengthen stomach and lung	Bulb
	*Ophiopogon japonicus*	Nourishing	Tuber	Smooth lung and nourish yin	Root
	*Dendrobium nobile*	Diabetes, improve eyesight, nourishing, promote digestion	Stem	Strengthen stomach and promote fluid production, nourish yin and clear heat	Stem
	*Plantago asiatica*	Heat-clearing and detoxifying, induce diuresis, eliminating phlegm	Whole plant	Clear heat and dampness, induce diuresis	Whole plant, seed
	*Fagopyrum dibotrys*	Heat-clearing and detoxifying, promote blood circulation, calculus	Root, rhizome	Clear heat and detoxifying	Rhizome
	*Artemisia argyi*	Gynaecopathia	Whole plant	Stop bleeding, expel cold and alleviate pain by warming meridians	Aerial part
Inconsistency in efficacy	*Fallopia multiflora*	Nourishing	Tuber	Moisten intestines and relax bowls	Tuber
	*Parthenocissus tricuspidata*	Rheumatism, promote blood circulation	Root, stem, fruit	Clear away heat and promote dieresis	Root, stem
	*Acorus tatarinowii*	Cold	Whole plant	Eliminate dampness and stimulate appetite, regain consciousness through dispelling phlegm, induce resuscitation and strengthen intelligence	Rhizome
	*Trachelospermum jasminoides*	Rheumatism	Whole plant	Clear heat and relieve toxicity	Aerial part
	*Hypericum japonicum*	Heat-clearing and detoxifying, promote blood circulation, promote digestion	Whole plant	Clear liver, promote diuresis to drain dampness and relieve dyspepsia	Whole plant
	*Leonurus artemisia*	Heat-clearing and detoxifying	Whole plant	Activate blood and dispel stasis, induce dieresis and alleviate edema	Whole plant

By comparing the herbal tea plants commonly used in Jianghua and Lingnan, there are 23 common plant species among which 6 species have different functions (Table 3). They are *Fallopia multiflora*, *Parthenocissus tricuspidata*, *Acorus tatarinowii*, *Trachelospermum jasminoides*, *Hypericum japonicum*, and *Leonurus artemisia*.

The coefficient of similarity of herbal tea plants commonly used in Jianghua and Lingnan is 11.2%. Using chi-square analysis, the number of mentions for part(s) used varied significantly between the two culturally distinct communities (*p* value < 0.05).

## Discussion

### Prospective value of herbal tea plants used by Yao people

Herbal tea in Lingnan region is based on the theory of traditional Chinese Medicine (TCM); many recipes used in herbal tea are evolved from prescriptions of TCM [17]. However, Yao people in Jianghua did not record their traditional knowledge of herbal tea with books or scripts instead of folksongs and teaching generations by experience and dictation. We compared herbal tea plant in Jianghua with Drug Standard Database (http://www.drugfuture.com/standard/), including Chinese PharmacopeiaI (2015 and 1977 versions), Tibetan medicineI, Uygur medicine, and Japanese Pharmacopoeia, and 124 species are not listed in Pharmacopeia (Table 1). Among these 124 species, the medicinal use of not all species can be supported by literatures. For example, Yao people in Jianghua indicated that *Achyranthes aspera* can relieve pain, which was verified by Barua et al. In 2010, they verified the antinociceptive activity of the methanolic extract of leaves of *A*. *aspera* in animal models of nociception [18]. *Cirsium japonicum* stops bleeding, which was verified by Chen Qi et al. in 2012 [19]. However, most of these 124 species cannot be found in the supporting literatures. Yao people in Jianghua generally believed that *Clematis henryi* is a good medicine for relieving pain, *Heteropanax fragrans* can treat rheumatism, and *Marsdenia sinensis* can treat heatstroke. There is a great need to further study these plant species.

### The efficacy and safety of species used in Jianghua

In Jianghua, heat-clearing and detoxifying is the most common medicinal function, followed by treating rheumatism, because the Dragon Boat Festival is at the end of spring and the beginning of summer, weather conditions are hot and humid, so the main plant materials used for herbal tea are focused on heat-clearing and detoxifying and treating rheumatism.

In Jianghua, 22 species were involved in eliminating inflammation; however, of the 83 species used for heat-clearing and detoxifying, 14 species were involved in eliminating inflammation; it shows that 63.6% of the medicinal plant species sold to eliminate inflammation are also used for heat-clearing and detoxifying, so it is important to conduct some studies to understand the dual effect and discover the possible relationship, which is useful for the theoretical construction of the traditional Chinese medicine (TCM).

Over the past 20 years, the safety [20] and pharmacological efficacy [21–24] of herbal drinks have drawn attention. Findings have elucidated that some phytochemicals in herbal tea are beneficial to human health [25–28], while some are risky to humans [29–34]. Therefore, further research is needed to analyze the bioactivity and toxicity of herbal tea. Among 169 species, two of them are forbidden as raw materials for food based on an announcement from The National Health Commission of the People’s Republic of China (http://www.nhfpc.gov.cn/). They are *Dysosma versipellis* (Hance) M. Cheng ex Ying and *Tripterygium wilfordii* Hook. f.

*Dysosma versipellis*: Podophyllotoxin, a chemical compound isolated from *D*. *versipellis*, is recorded to show cytotoxicity resulting emesis, diarrhea, and hepatic and central nerve system lesion [35–38]. However, due to its chemical function similar to colchicine, podophyllotoxin and its derivatives have been synthesized and utilized as anti-tumor drugs [39]. Besides, it was recorded to be used as an antiviral material for treating condyloma acuminatum caused by human papilloma virus (HPV) [40]. *D*. *versipellis* is largely be utilized for clearing heat and detoxification, rheumatism, and promoting blood circulation by Yao people in Jianghua. However, due to excessive consumption, the conservation status of *D*. *versipellis* on “China’s red list” is registered as vulnerable. At present, *D*. *versipellis* is cultivated in Jianghua.

*Tripterygium wilfordii*: The extract of *T*. *wilfordii*, a Chinese herb, has anti-inflammatory and immunosuppressive activities and an established history of use in the treatment of rheumatoid arthritis [41, 42]. However, the most common side effects of *T*. *wilfordii* are gastrointestinal tract disturbances, such as diarrhea, leukopenia, thrombocytopenia, rash, skin pigmentation, and malfunction of the male and female reproductive system [43].

### Comparison of plant materials used for herbal tea in Jianghua and Lingnan

The resurgence of interest in natural products has fueled the global herbal tea market. In 2013, Yujing Liu recorded 241 species used for herbal tea in Lingnan Region (China) [1]. By comparing the herbal tea plants commonly used by Jianghua and Lingnan, there are 23 common plant species, among which, there were 17 species that had consistent function and 6 species have different functions.

By comparing the 6 species having different functions in Jianghua and Lingnan, we cannot confirm that they have various medical functions. *Achyranthes aspera*, *Fagopyrum dibotrys*, *Lonicera confuse*, *Lonicera japonica*, *Dendranthema morifolium*, and *Juncus effusus* are heat-clearing and detoxifying herbs. In Chinese medicine, the lower the fire is equal to clear heat. We found that there may be a relationship between detoxifying and antibacterial or anti-inflammation properties, because most of the plants with detoxifying properties have antibacterial or anti-inflammation effects (Table 3) [44–52]. For *Gleditsia sinensis*, Jianghua people pointed that it can induce diuresis, and the Lingnan people indicated that it can relieve pathological heat and remove dampness through diuresis. This may represent a direction for our study of the activity of Chinese herbs. So it will be necessary to verify the pharmacological activity in the future.

By comparing the herbal tea plants commonly used by Jianghua and Lingnan, the coefficient of similarity of herbal tea plants is 11.2%, which is low. We compared all plant parts used in the Jianghua and Lingnan regions. The common used plant parts are whole plant, root, leaf, stem, rhizome, tuber, flower, fruit, bark, seed, pith, branch, and shoot thorn. In Lingnan region, there are several particular used plant parts. They were aerial part, bulb, kernel, bud, peel, stigma, stem node with horns, and pollen. However, in Jianghua region, the particular used parts are pod, shoot, and fruit cluster. We selected all common used parts to do statistical analysis with chi-square analysis; the results (*p* value < 0.05, *χ*^2^ = 61.333) show the used plant parts varied significantly between these two different regions. Hence, the variation of used plant part in two regions accounts not only for the particular mentioned used parts but for varied usage rate of each common used part. For example, in Lingnan region, root (20.78%) is the most frequently mentioned used part, while in Lingnan region, it is whole plant (38.36%). The variation of plant part used suggests that the medical plant tradition is far different between the Lingnan and Jianghua regions. The low coefficient of similarity and the variation of plant part used reflect a relatively great difference of herbal tea plant tradition between Jianghua and Lingnan.

### The traditional medicinal market is a bit unstructured

In the ethnobotanical surveys, we found that there are 14 poisonous species, which need to be payed attention. They are *Pothos chinensis* (Raf.) Merr., *Typhonium flagelliforme* (Lodd.) Blume, *Trachelospermum jasminoides* (Lindl.) Lem., *Asarum sagittarioides* C. F. Liang, *Dysosma versipellis* (Hance) M. Cheng ex Ying, *Celastrus orbiculatus* Thunb., *Tripterygium wilfordii* Hook. f., *Senecio scandens* Buch.-Ham. ex D. Don, *Hemsleya macrosperma* C. Y. Wu ex C. Y. Wu et C. L. Chen, *Reineckia carnea* (Andr.) Kunth, *Eomecon chionantha* Hance, *Fallopia multiflora* (Thunb.) Harald., *Stemona tuberosa* Lour., and *Schizocapsa plantaginea* Hance. In addition, we do not know if there is a phenomenon of substitutes or adulterants in Jianghua traditional market. Based on the Drug Standard Database, we listed the originality of all of the species (Table 1). So, the plants that are nonexistent in the Drug Standard Database need to be scientifically investigated for their efficacy and safety in the future.

## Conclusions

The traditional medical market in Jianghua Yao Autonomous County reflects the plant species richness and cultural diversity. Traditional knowledge of herbal tea is the result of the accumulated experience by the Yao people’s long-term struggle against disease, so many aspects must be scientific. With the rise of natural product drugs, there is the need to analyze the chemical composition and activity of the materials of herbal tea. Future research is also needed to understand the safety and efficacy of the recorded herbal tea. For sustainable utilization, the production of herbal tea should be monitored.

In addition, uniform standards of practice and licensing of herbal vendors is required to produce a safer herbal tea market. It is very important for them to have the knowledge to select the proper plants since some herbs are hard to identify due to similar morphological characteristics.

## Abbreviations

CS: Cognitive salience
LC: Least concern
NE: Not evaluated
S: Coefficient of similarity
UV: Use value
VU: Vulnerable
